# Assessment of the bypass of obturation materials beyond fractured instruments after using different obturation techniques in simulated curved canals (An in-vitro study)

**DOI:** 10.1371/journal.pone.0318095

**Published:** 2025-01-24

**Authors:** Ammar Aziz Lateef, Mohammed Rasheed Hameed, Noor Hayder Fadhil, Ahmed Hamid Ali

**Affiliations:** Aesthetic and Restorative Dentistry Department, College of Dentistry, University of Baghdad, Baghdad, Iraq; Universidade Federal Fluminense, BRAZIL

## Abstract

This study evaluated the extent to which obturation materials bypass fractured endodontic instruments positioned in the middle and apical thirds of severely curved simulated root canals using different obturation techniques. Sixty resin blocks with simulated root canals were used, each with a 50° curvature, a 6.5 mm radius of curvature, and a length of 16.5 mm, prepared to an ISO #15 diameter and taper. Canals were shaped using ProTaper Universal files (Dentsply Maillefer) attached to an X-smart Plus endo motor (Dentsply), set at 3.5 Ncm torque and 250 rpm, up to size S2 at working length. To simulate fractures, F2 and F3 files were weakened 3 mm from the tip, then twisted to break in the apical and middle sections of the canal, respectively. All samples were sealed with GuttaFlow 2 and divided into three groups (n = 20/group) according to obturation technique: A) single cone, B) lateral condensation with a rotary spreader, and C) softcore obturators. Each group was then divided into two subgroups (n = 10) based on the instrument fracture location (1 = apical, 2 = middle). The linear intrusion of obturation materials through the fractured instruments was measured using ImageJ software and analyzed statistically with ANOVA, Tukey tests, and independent t-tests, with significance set at p<0.05. Material bypass in group B1 (3.27 ± 0.63 mm) was significantly greater than in group A1 (2.39 ± 0.44 mm) and group C1 (2.91 ± 0.77 mm). In group C2, bypass (5.76 ± 0.64 mm) was significantly higher than in group A2 (3.82 ± 0.2 mm) and group B2 (2.27 ± 0.96 mm). Additionally, bypass in group A2 was greater than in group B2, and group B1 had more bypass than B2, while group C2 exceeded C1. The lateral condensation technique with a rotary spreader and softcore obturators increased the bypass of obturation materials through fractured instruments in simulated curved canals. These techniques may thus enhance material flow in endodontic procedures involving instrument fractures.

## Introduction

Endodontics treatment aims to provide adequate cleaning and shaping of the canals to eliminate the microbial load and provide a hermetic seal, this should be done by a skilled physician who knows how to deal with tight spaces and precise instruments so the experience could develop a tactile awareness allowing to detect the increase in frictional stress to avoid complications and how to manage if happened [1], during cleaning and shaping instrument fracture might occur and that ranging between 0.28–16.2% [2, 3], teeth factors have a major role affecting on fracture ratio as in anterior teeth 0.28%, in premolars 1.56% and molars 2.74% [4], the prevalence of instrument fracture at the apical third 52.5%, at the middle third 27.5%, and the coronal third is 12.5% [2], considering radius of curvature, canal with 5 mm radius of curvature has lower cyclic fatigue of instrument to fracture than canal with 10 mm radius [5].

The outcome of endodontic treatment with instrument fracture depends on whether the tooth was vital or not and the stage of cleaning and shaping in infected teeth [6], there are different ways of treating teeth with fractured instrument including first, removal of the broken instrument using modified Gates Glidden burs and different ultrasonic tips under operating microscope with the aid of special grip instruments like Masserann kits, IRS and others [7, 8], this method requires the removal of sound dentine leading to weakening of root strength and increase the possibility of vertical fracture in future [9], root perforation is another consequence that might happen during instrument removal especially in the curved canal so this should be taken into consideration before proceeding the treatment [10].

Second, bypassing the separated instrument which is considered the safest method that does not require the removal of excessive dentine tissue from the root canal allowing instrumentation and obturation up to the apex, using a pre-curved k-file #10 at the edge with an attempt to advance it between the broken piece and the canal wall using slight pressure quarter of a turn with lubricant inside and frequent irrigation till completely pass the fragment, radiograph should be taken during this procedure to avoid perforation [11, 12], rotary instrument is not recommended to be used for broken piece removal to avoid new separation [13].

Lastly, Entombing the fractured piece, in vital pulp cases when the fractured piece is apically located or beyond the curvature the best decision is to obturate over it with warm gutta percha to avoid removing of root dentin, in case of an infected canal it depends on the stage of cleaning and shaping, if it was at the final and the fragment located at the apical or middle third, thorough irrigation should be done with 5.25 NaOCl followed by 17% EDTA and finally with 2% chlorhexidine with copious normal saline irrigation between them to avoid interaction [14, 15], then obturation done over the fragment, if the fracture happened at early stage beyond the curvature or at the apex and the bypass or removal is difficult without removing excessive dentine tissue, continue cleaning and shaping of the canal wall above the fragment then dressing with CaOH for possible disinfection is recommended for one month followed by obturation and constant follow up for 6–12 months, if there was any sign or symptoms then apical surgery might be considered [8, 16].

This study aimed to assess the extent of bypass of obturating material beyond fractured instruments in severely curved simulated root canals after obturation with single cone, lateral compaction, and softcore obturation techniques. The null hypothesis stated that there is no difference in the extent of the obturation materials beyond the fractured instruments between different techniques or between middle and apically positioned fractured instruments.

## Material and method

### Simulated canals and grouping

Power calculations were performed using G*Power version 3.1.9.6 (Franz Faul, Universitat Kiel, Germany), a study power of 95% power, 5% alpha error, and an effect size of 0.9 [17], 48 samples were needed in total (n = 8/group). Ten samples were used for each group.

**Fig 1:**
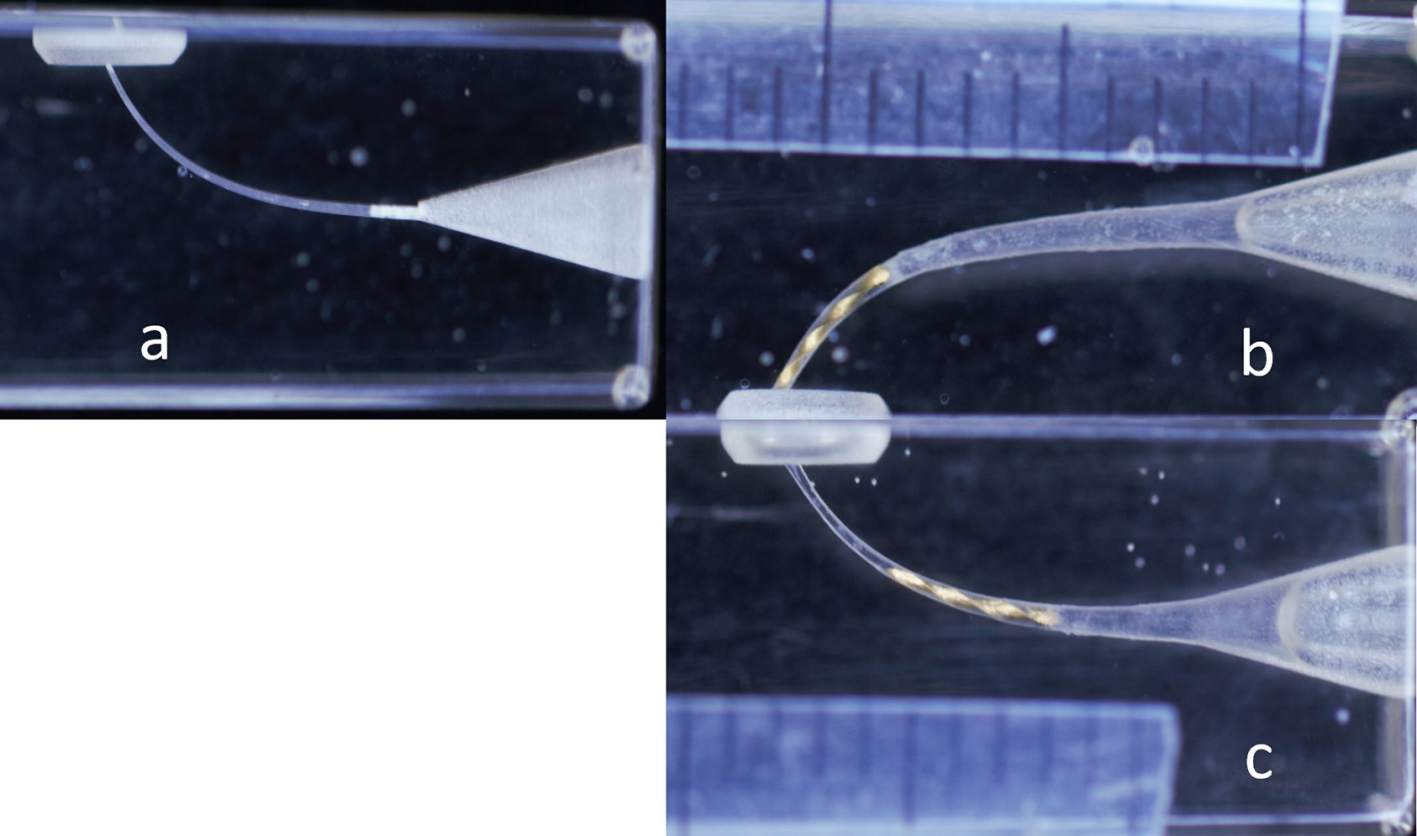
Simulated canal resin blocks a: Initial stage of simulated canal resin block before instruments fracture, b: Apical fractured instrument, c: Middle fractured instrument.

Sixty simulated single curved root canal resin blocks were used (Dentsply -Maillefer, Balaigues, Switzerland) with a length of 16.5 mm, taper, and diameter of ISO #15 instrument with a curve of 50 degrees, and radius of curvature of 6.5 mm, samples were divided into six groups each of 10 according to the position of instrument fracture (in the apical or middle section of the root canal, as shown in" Fig1") and the method of obturation used.

Groups of the study included, A1/ Canals with apically fractured instruments obturated using the single cone technique. A2/ Canals with middle fractured instruments obturated using the single cone technique. B1/ Canals with apically fractured instruments obturated by lateral condensation using a rotary spreader corresponding to size #25 k-file held by a handpiece of a programmed endodontic engine with reciprocation motion (X smart plus; Dentsply, Switzerland). B2/ Canals with middle fractured instruments obturated by lateral condensation using the rotary spreader mentioned above. C1/ Canals with apically fractured instruments obturated by thermoplasticized Gutta Percha (softcore obturator). C2/ Canals with middle fractured instruments obturated using softcore obturator.

### Preparation of the canals

The canals were instrumented using Protaper universal NiTi rotary files (Dentsply Maillefer, Switzerland) in the following sequence (S1, S2, F1, F2, F3), and an endo motor (X smart plus, Dentsply, Switzerland) set at 250 pm and 3.5 torque.

To have the instrument fracture at the apical part of the canal, Sx was excluded because of the shape of the coronal part of the resin block, preparation started with S1, and S2 files to a 15 mm working length (standardized endodontic preparation), irrigation with isopropyl alcohol before and after each file for 15sec with recapitulation using k file #10 to remove derbies [17], continue preparation with F1 file shorter 3 mm from WL, F2 file was nicked at 3mm from the tip using a flat disc diamond bur, then introduced into the canal to have that part to engage and fracture at the apex. For the middle fracture instrument, the canal was prepared as previously mentioned, but the F1 file was introduced 6mm shorter than WL, F2 was skipped to the F3 file that was nicked at 3 mm from the tip and held into the canal to engage and fracture at the middle third, as shown in "Fig 1B and 1C".

In this study the distance from the simulated canal apical foremen to the coronal end of the fractured instrument was measured, First the resin blocks were fixed on a base with a standardized distance from a stereomicroscope (Leica MZ 12.5, Heerbrugg, Germany), for an accurate measurement digital images were taken with a scaled ruler beside the blocks and by using image J program these distances were measured in mm.

### Obturation of the canal

**Fig 2:**
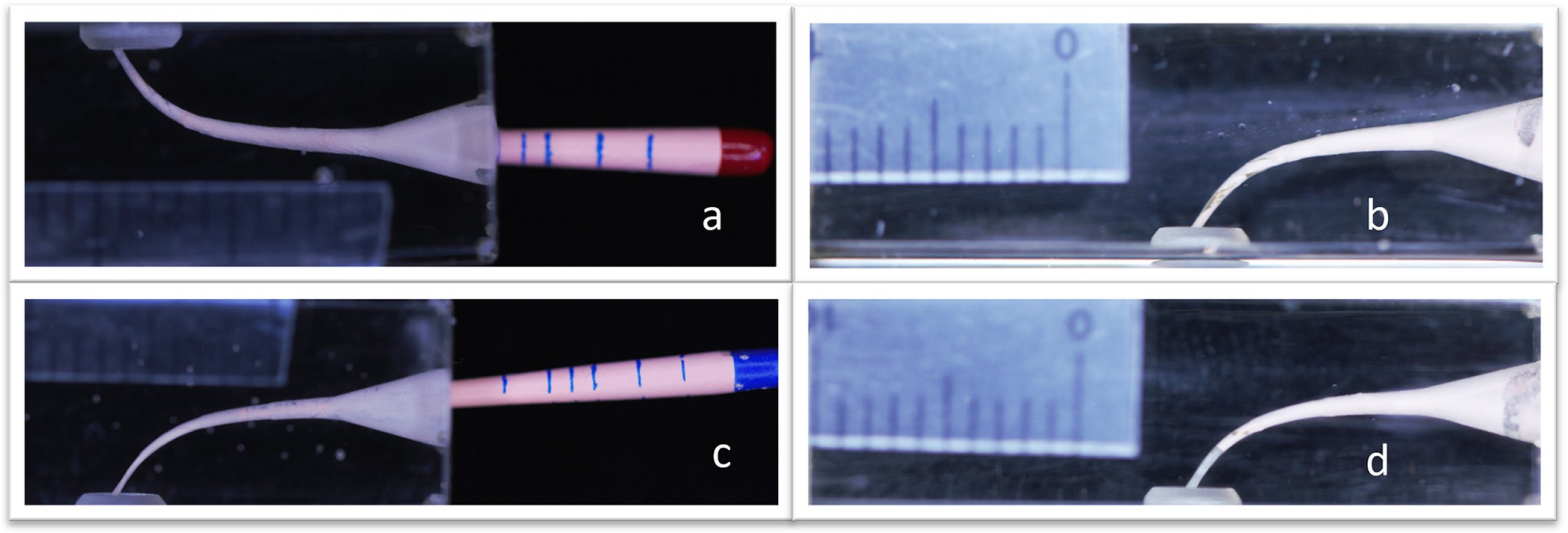
Single cone obturation, a and b: Apical fracture instrument obturation, c and d: Middle fracture instrument obturation.

**Fig 3:**
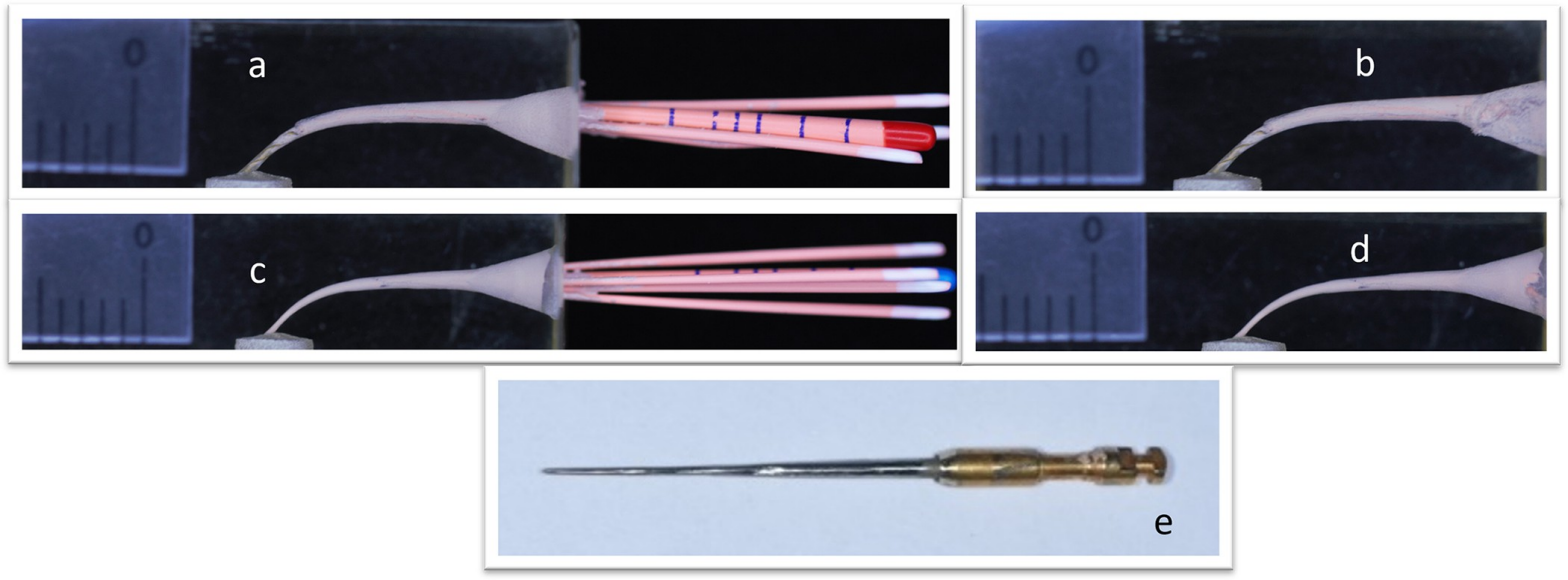
Lateral obturation, a and b: Apical fracture instrument obturation, c and d: Middle fracture instrument obturation, e: NiTi rotary spreader.

Obturation procedure was performed using Gutta Flow2 sealer (Coltene/Whaledent) for all groups of this study, for A1 group F2 point (Dentsply Maillefer) cut 3 mm from the tip using a scalpel blade on a glass slab to have a straight cut to fit over the fractured piece with a tug back, a fresh amount of sealer was mixed for each sample to maintain physical and chemical properties then applied into the canal by a lentulo spiral size# 25 and obturation performed with the modified F2 gutta percha point, cut the excess of GP with thermacut at the canal orifice then condense with a plugger using a load of 1 kg for 60 s determined by an electronic balance weight [18]. The obturation method is shown in "Fig 2A" for the A2 group, the F3 GP point was cut 6 mm from the tip to fit over the broken piece and the obturation was done as in group A1" Fig 2B". For B1 and B2 groups F2 and F3 GP points were used respectively for apical and middle fracture with the use of a specially designed NiTi rotary spreader [17] and GP auxiliaries ISO # 15 to fill the canals, cut the excess at the canal orifice with a thermacut and condense with a plugger as mentioned previously, as shown in "Fig 3".

**Fig 4:**
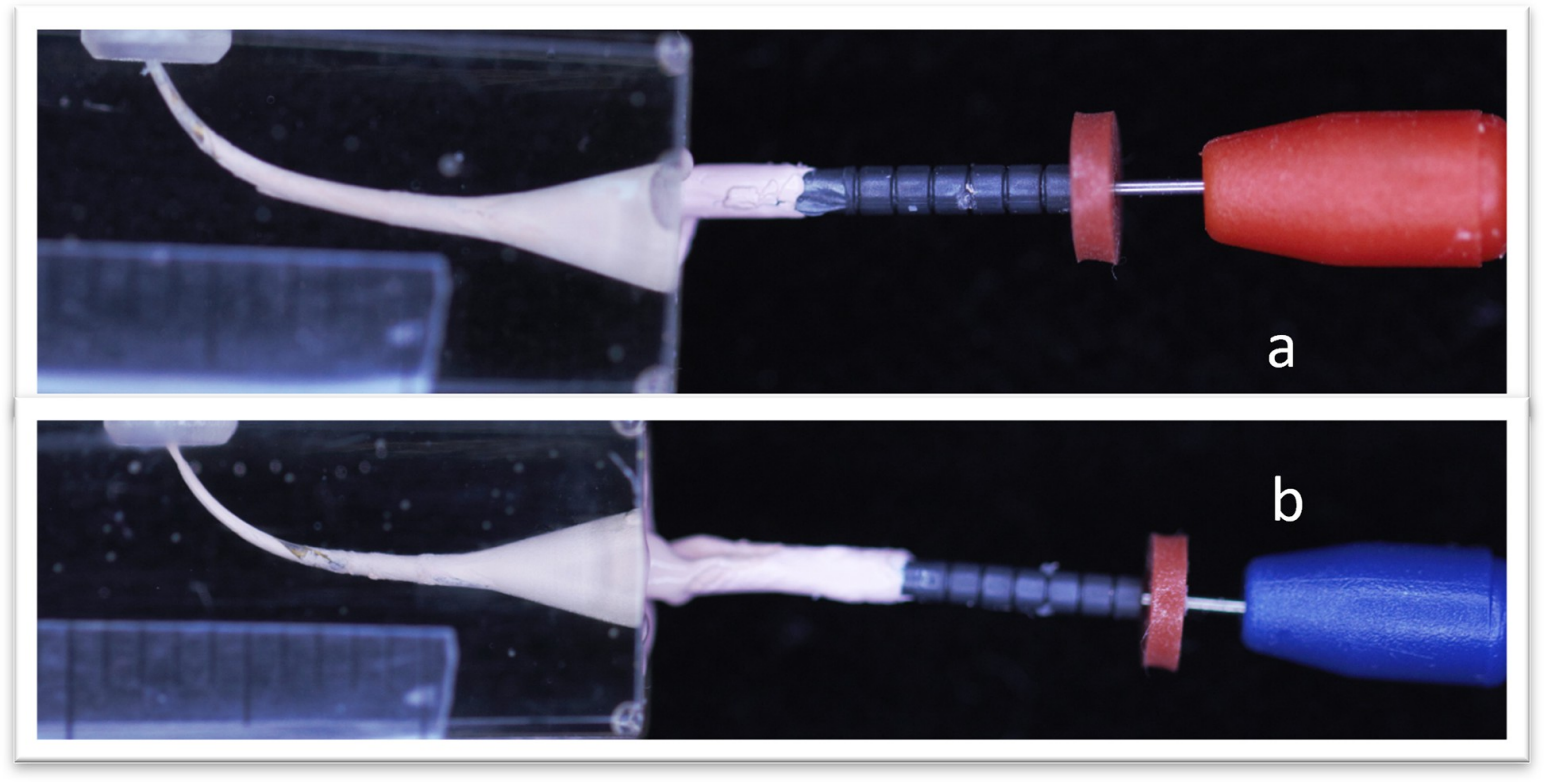
Softcore obturation, a: Apical fracture instrument obturation, b: Middle fracture instrument obturation.

For C1 and C2 groups soft core obturators equivalent to ISO # 25 and 30 (CMS Dental A/S, Roslev, Denmark) were used for apical and middle fractured-piece canals respectively, the obturator’s tips were cut as in group A1, and A2, then heated in the softcore DT oven (CMS dental, Njalsgade 21G.Dk-2300 Copenhagen, Denmark) and after application of the sealer, a heated core was introduced into the canal under a steady load (1kg), then cut the surplus at the canal orifice, as shown in "Fig 4".

To measure the apical bypass of obturation materials around the fractured instrument, the distance from the simulated canal apical foremen to the apical extension of obturation materials (D) was measured for all samples by taking digital images and analyzed. The apical bypass of the obturation materials beyond the fractured instrument was measured by subtraction of D measurement from that distance between the simulated canal apical foremen to the coronal end of the fractured instrument. The statistical tests used in this study included ANOA, Tukey test, and paired t-test. Statistical significance was at p<0.05.

## Results

### Apical broken instrument bypass by obturation materials

**Fig 5:**
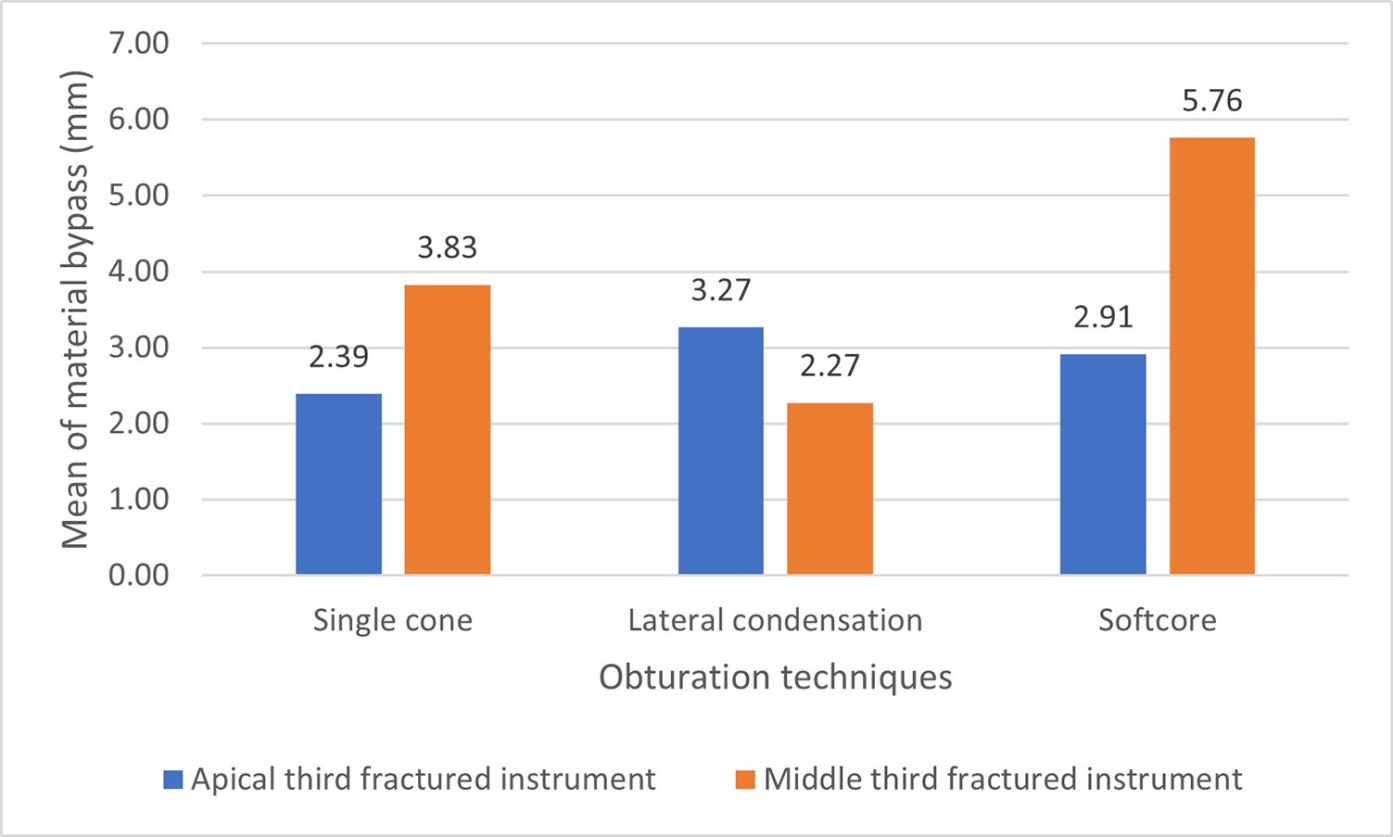
Bar chart of the means of bypass by the obturating materials in canals with middle and apically positioned fractured instruments.

This study showed that group B1 has the highest mean value (3.27±0.63), While group A1 has the lowest mean value (2.39±0.44), as shown in "Fig 5".

The results showed that there were statistically significant differences between the groups (p<0.05). Multiple comparisons showed that the bypass of fractured instruments by the obturating materials after obturation with lateral condensation using the rotary condenser was significantly higher than after the single cone and the softcore obturation techniques, respectively (p<0.05). Also, there was no significant difference between the single cone and the softcore obturation techniques (p>0.05), as shown in "Table 1".

**Table 1:** Mean, minimum, and maximum of the bypass of obturating materials beyond the fractured instrument in the simulated canals in different study groups.

Groups	N	Mean (mm)	Standard Deviation	95% Confidence Interval for mean		Minimum	Maximum	p-value*
				Lower Bound	Upper Bound			
A1	10	2.39^a^	0.44	2.08	2.70	1.84	3.24	0.015
B1	10	3.27^a,b,A^	0.63	2.81	3.72	2.41	4.38	
C1	10	2.91^b,B^	0.77	2.36	3.46	1.54	4.14	
A2	10	3.83^b,c^	2.01	2.39	5.26	0.44	6.62	<0.001
B2	10	2.27^b,d,A^	0.96	1.58	2.96	1.08	3.86	
C2	10	5.76^c,d,B^	0.64	5.30	6.22	4.59	6.51	

*One-way ANOVA between groups, Identical superscript lowercase letters represent significant differences between the relevant groups (Tuckey posthoc, p<0.05). Identical superscript uppercase letters represent significant differences between the relevant groups (Independent sample t-test, p<0.05).

### Middle broken instrument bypass by obturation materials

The result of this study showed that group C2 has the highest mean value (5.76±0.64), While group B2 has the lowest mean value (2.27±0.96), as shown in "Fig 5. The result showed that there were statistically significant differences among the groups (p<0.001). Softcore obturation has a statistically significantly higher bypass of obturation materials than that of lateral condensation single cone obturations, respectively (p<0.05). Also, the single cone obturation has a statistically significantly higher bypass of obturation materials as compared with the lateral condensation obturation (p<0.05).

Independent Sample t-test showed that group B1 has a statistically significantly higher bypass length of obturating materials than group B2 (P = 0.014), also the group C2 has a statistically significantly higher bypass length of obturating materials than group C1 (P<0.001), while there was no statistically significant difference between group A1 and group A2 (P = 0.052).

## Discussion

Successful endodontic treatment requires thorough cleaning and shaping of the canals mechanically and chemically with a proper 3D obturation to provide a hermetic seal against microbial infusion coronally and apically [19], NiTi files have been developed to ease canal preparation, yet instrument fracture creates a major problem during routine endodontic treatment, numerous factors play a role in that including operator’s skill, instrument design, and manufacturing, cyclic fatigue, root canal anatomy and configuration [20]. Postgraduate students have a higher risk to fracture instruments compared to undergraduate students because they utilize rotary endodontic files more frequently in preparation of root canals which exhibit both torsional and cyclic fatigue compared to traditional stainless steel files [21].

Considering manufacturing design, taper, and processing instruments produced by twisting have a greater cyclic fatigue resistance than those produced by grinding [22], files with triangular cross-sections have better fatigue resistance than those with square cross-sections [23], instruments with large diameters have a higher incidence of fracture around a curve [24]. According to metallurgical characteristics, instruments made from M wire reveal more flexibility and resistance to cyclic fatigue than conventional NiTi files, M wire contains an austenite phase with a small amount of martensite, and R phases in which the austenite phase is responsible for the strength of the wire [25], stainless steel files show signs of fatigue before fracture while NITI files are flexible and didn’t show any signs of fatigue before fracture [26], electropolishing of the instrument during manufacturing increase the resistance to cyclic fatigue by reducing the surface irregularities that serve as points of stress and crack initiation [27].

Simulated canals in resin blocks have been used as substitutes for extracted human teeth for pre and post-instrumentation standardization [28]. Separation of an instrument at the beginning of treatment affects the prognosis negatively, but if the separation happens at the last stage or in an aseptic root canal, then the prognosis is better [16]. Instrumentation and obturation coronal to the broken file was one of the options especially when there is a thin L-shaped root with the fractured piece at or beyond the curvature and there is a great danger of having perforation when attempting to bypass or remove the fractured piece [8].

For apically fractured file, it has been found that obturation with lateral condensation using rotary spreader group (B1) had higher bypass of obturation materials apical to the coronal end of the broken piece than that after single cone (A1) and soft core (C1) groups, in studies done by Mohammed R et al. (2015, 2018) [17, 29] comparing the use of finger spreader and reciprocating NiTi rotary spreader with lateral condensation technique to obturate lateral canals, they found that there were a higher extension of obturation materials into coronal and apical lateral canals with the use of a reciprocating NiTi rotary spreader, they assumed that the engine driven rotary spreader may create mechanical friction and heat generation which could plasticize the Gutta Percha increasing its flowability and extrusion together with the sealer into lateral canals, beside all sealers have thixotropic and pseudoplastic properties when subjected to constant or increased shear stresses respectively during lateral condensation [30, 31]. Also, in studies done by Blum JY et al. 1998 [32a, 33b] they found that vertical forces obtained by lateral condensation were almost six times the force exerted with thermafil and single cone techniques, these findings could explain the reason for more bypass of obturation materials with lateral condensation technique using rotary spreader in canals with apically fractured instruments in this study.

It has been found that the 3mm fractured instrument at the apical third did not influence the time required for bacterial penetration, besides the presence of flutes in the files and the radicular cross-section in natural teeth as it is not exactly round or ovoid that permit the extrusion of obturation materials to incorporate with the fractured piece as one unit filling material [16, 34].

For the middle fractured instrument, the softcore obturation group (C2) had more apical bypass of obturation materials than single cone (A2) and lateral condensation (B2) groups, this could be attributed to the thixotropic behavior of thermoplasticized Gutta Perch which lower the viscosity and increase the insertion rate [31, 34–36], beside the shorter canal length could affect the extrusion of obturation materials in A2, B2 groups.

Another finding of this study was that group B1 had a higher bypass of obturation materials than group B2, in studies done by R. Fernandez et al. and C. K. Teixeira et al. in 2016 and 2017, respectively [37, 38], they found that sealers have lower penetration into coronal lateral canals than into apical lateral canals, during lateral condensation the shear stress is instant and not continuous, and this force is dissipated by decreasing the pressure on the wall of the canal by increasing the taper coronally and losing the mass of the obturation materials resulting in a low flow of the sealer into coronal lateral canals, beside the heat generated by the friction of NiTi spreader could plasticize more of the gutta percha at the apical part than the middle part of the canal [29], explaining the more extrusion of obturation materials with apical fracture instrument than middle fracture using lateral condensation with reciprocating NiTi spreader. In cases with internal root resorption, the pressure created by the force of obturation using the continuous wave compaction or lateral condensation with Bio-C repair sealer plays a significant factor in obtaining the maximum obturation volume compared to the obturation with bulk-fill with Bio-C Sealer alone [39].

Also, it has been found that group (C2) had more bypass of obturation materials than the (C1) group, this could be because PTU files produce a great modification on the shape of the canal with a tendency to straighten the curvature and widen the canal, especially at the middle third [40–42].

The limitations of this study include the use of simulated root canal resin blocks, a single endo-file system, one type of endodontic sealer, and the use of linear measurements rather than 3D volumetric assessments of the obturation materials.

## Conclusion

It has been found that the obturation with a thermoplasticized GP and GuttaFlow2 sealer improves the bypass of obturation materials apical to the coronal end of the fractured instrument, and the use of reciprocating NiTi spreader with lateral condensation technique improves the bypass of obturation materials apically.
